# Employing Molecular Conformations for Ligand-Based Virtual Screening with Equivariant Graph Neural Network and Deep Multiple Instance Learning

**DOI:** 10.3390/molecules28165982

**Published:** 2023-08-09

**Authors:** Yaowen Gu, Jiao Li, Hongyu Kang, Bowen Zhang, Si Zheng

**Affiliations:** 1 Institute of Medical Information (IMI), Chinese Academy of Medical Sciences and Peking Union Medical College (CAMS & PUMC), Beijing 100020, China; yg3191@nyu.edu (Y.G.); li.jiao@imicams.ac.cn (J.L.); kang.hongyu@imicams.ac.cn (H.K.); 2 Department of Chemistry, New York University, New York, NY 10027, USA; 3 Department of Biomedical Engineering, School of Life Science, Beijing Institute of Technology, Beijing 100081, China; 4 Beijing StoneWise Technology Co., Ltd., Beijing 100080, China; zhangbowen@stonewise.cn; 5 Institute for Artificial Intelligence, Department of Computer Science and Technology, BNRist, Tsinghua University, Beijing 100084, China

**Keywords:** virtual screening, bioactivity prediction, equivariant graph neural network, multiple instance learning, molecular conformation, benchmark dataset

## Abstract

Ligand-based virtual screening (LBVS) is a promising approach for rapid and low-cost screening of potentially bioactive molecules in the early stage of drug discovery. Compared with traditional similarity-based machine learning methods, deep learning frameworks for LBVS can more effectively extract high-order molecule structure representations from molecular fingerprints or structures. However, the 3D conformation of a molecule largely influences its bioactivity and physical properties, and has rarely been considered in previous deep learning-based LBVS methods. Moreover, the relative bioactivity benchmark dataset is still lacking. To address these issues, we introduce a novel end-to-end deep learning architecture trained from molecular conformers for LBVS. We first extracted molecule conformers from multiple public molecular bioactivity data and consolidated them into a large-scale bioactivity benchmark dataset, which totally includes millions of endpoints and molecules corresponding to 954 targets. Then, we devised a deep learning-based LBVS called EquiVS to learn molecule representations from conformers for bioactivity prediction. Specifically, graph convolutional network (GCN) and equivariant graph neural network (EGNN) are sequentially stacked to learn high-order molecule-level and conformer-level representations, followed with attention-based deep multiple-instance learning (MIL) to aggregate these representations and then predict the potential bioactivity for the query molecule on a given target. We conducted various experiments to validate the data quality of our benchmark dataset, and confirmed EquiVS achieved better performance compared with 10 traditional machine learning or deep learning-based LBVS methods. Further ablation studies demonstrate the significant contribution of molecular conformation for bioactivity prediction, as well as the reasonability and non-redundancy of deep learning architecture in EquiVS. Finally, a model interpretation case study on CDK2 shows the potential of EquiVS in optimal conformer discovery. The overall study shows that our proposed benchmark dataset and EquiVS method have promising prospects in virtual screening applications.

## 1. Introduction

Virtual screening [1] adopts computational methods to identify chemical candidates that may have binding bioactivities to a query target, which is widely used in the early stage of drug discovery [2,3]. There are two main categories of VS: structure-based VS (SBVS) and ligand-based VS (LBVS). LBVS methods generally predict unknown bioactivities of new molecules based on known bioactivities of molecules. The commonly used methods of LBVS are pharmacophore mapping [4], shape-based similarity [5], fingerprint similarity [6], and machine learning-based Quantitative Structure–Activity Relationship (QSAR) [7,8,9]. The main assumption of these methods is that “structurally similar molecules have similar bioactivities for specific targets” [10]. Following the assumption, as there are good consistencies of structure differences and bioactivity differences, chemical knowledge (molecular fingerprints) and representations (molecular embeddings) extracted from known molecular structures can be used for bioactivity prediction.

With the massive development of public pharmaceutical databases and artificial intelligence technologies, data-driven deep learning frameworks have been widely used in the vast majority fields of drug discovery, such as de novo drug design [11,12], ADMET prediction [13,14], drug repositioning [15,16], and VS [17,18]. Compared to traditional LBVS methods, deep learning-based methods map molecular representations into high-dimensional spaces, and implicitly identify molecular similarities and correlations to bioactivities based on high-order embedding, which can be regarded as a continuous way to discover bioactive groups better than those traditional and discrete ones. Recently, there are several studies constructing LBVS and bioactivity prediction models using deep learning, where part of them are listed in Table 1. For instance, DeepScreening is a platform which uses deep neural networks trained from molecular fingerprints to predict molecular bioactivity values and categories [19]; GATNN is a graph neural network (GNN) framework extracts molecular fingerprints for similarity calculation in LBVS, which outperforms traditional fingerprints [20]; RealVS adopts a graph attention network (GAT) to learn molecule features from molecular graphs and optimizes multiple training loss with adversarial domain alignment and domain transferring for bioactivity prediction [21]. They experimentally proved that deep learning framework showed better structure representation ability than 2D structure-based molecular fingerprints.

However, the molecular 3D structures-molecular conformers, have seldom been considered in these studies. Previous studies have proven that there were certain relations between different molecular conformers and bioactivities [26,27,28], and several traditional fingerprint-based QSAR methods have already adopted molecular three-dimensional features extracted from molecular conformers for LBVS [29,30,31,32]. Therefore, employing molecular conformation for molecular representation learning is a promising strategy for bioactivity prediction. However, the available ***bioactivity prediction benchmark dataset with multiple molecular conformers*** is still lacking, and the ***end-to-end deep learning architecture*** that effectively learns molecular representations from molecular conformers has not been designed and estimated.

To address the above challenges, we first constructed a large-scale molecular bioactivity benchmark dataset with over 3 million endpoints, including nearly 1 thousand targets, 1 million molecules, and 10 million calculated molecular conformers. Then, we proposed a new deep learning method called EquiVS to introduce molecular conformation information into LBVS, which effectively improved molecular bioactivity prediction. EquiVS was designed as an end-to-end architecture with graph convolutional network (GCN), equivariant graph neural network (EGNN), and deep multiple instance learning (MIL) layers, which can directly learn molecular high-order representations from the 2D topological level and 3D structural level. The model performance comparison results on large-scale benchmark datasets indicated that EquiVS outperformed multiple classical machine learning-based and graph neural network-based baseline methods. Furthermore, the ablation study emphasized that efficient representations and attention-based aggregations of multiple molecular conformers play an important role in the accurate bioactivity prediction in EquiVS. To enhance the interpretability of EquiVS in real LBVS scenarios, we introduced an attention-based mechanism in deep MIL for optimal molecular conformer discovery which was investigated by a case study.

## 2. Results

### 2.1. Benchmark Dataset Quality Analysis

**Figure 1:**
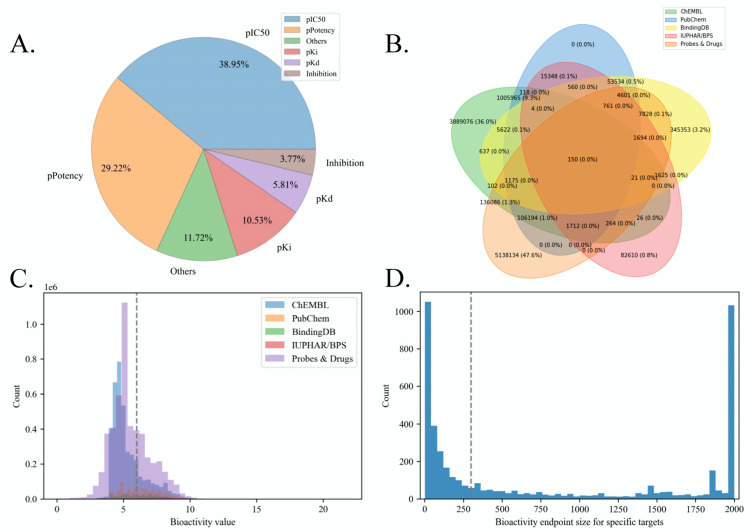
Distribution visualization of raw bioactivity endpoints. A. Pie plot of bioactivity type proportion.

We examined the quality of our constructed bioactivity benchmark dataset through visualization analysis. Extensive chemical structure (frequently occurred scaffolds, chemical spaces, etc.) and bioactivity distribution (bioactive molecule proportions, protein families, etc.) analysis for the initial source bioactivity data can be found in [33]. In this study, we focus on the data quality analysis of our re-curated data. Firstly, regarding the bioactivity unit, there are 1078 units in the initial integrated data, including pIC50 (38.95%), pPotency (29.22%), pKi (10.53%), pKd (5.80%), Inhibition (3.77%), pEC50 (3.05%), Activity (1.33%), pAC50 (1.24%), etc. Figure 1A shows the distribution of major bioactivity types in our raw data. It is suggested that our adopted five bioactivity types (pIC50, pPotency, pKi, pKd, and pEC50) make up the majority of the data (87.56%). Figure 1B showed the overlap between different source databases, indicating that only 12.4% of bioactivity endpoints are duplicated, and the majority of the raw endpoints are unique (e.g., 5.13 million unique data from Probe and Drugs and 3.88 million unique data from ChEMBL, with a proportion of 83.6% among overall endpoints). Therefore, these non-redundant bioactivity endpoints from different sources should be integrated to expand the data scale and promote bioactivity prediction.

Meanwhile, Figure 1C visualized the bioactivity value distribution among different source data. We set 1μM (6 -logM) as the threshold to classify active/inactive molecules, and the corresponding specific distribution results were listed in Table 2. The above results showed that the standard deviations of bioactivity values (within-group differences) among 5 source databases are close. The majority of bioactivity endpoints in ChEMBL and Probes&Drugs are inactive, while the proportions of active bioactivity endpoints in PubChem, BindingDB, and IUPHAR/BPS are larger than those of inactive ones. Considering between-group differences, the average bioactivity value differences between different source databases are acceptable (differences between average values are less than 1 -logM among ChEMBL, PubChem, BindingDB, and IUPHAR/BPS, which contains 99.2% of the overall endpoints). The above findings proved the reasonability of data integration from those source databases to expand the bioactivity benchmark data scale.

As for the selection of candidate targets, Figure 1D showed the distribution of bioactivity data scale of different targets in our benchmark dataset. It suggested that a considerable number of targets are with insufficient bioactivity endpoints (less than 100). Therefore, setting 300 as the data scale can help to filter the targets on which are more possible to construct low-capacity LBVS models, thus improving the quality of the benchmark dataset and the reliability of LBVS models for specific targets.

**Figure 2:**
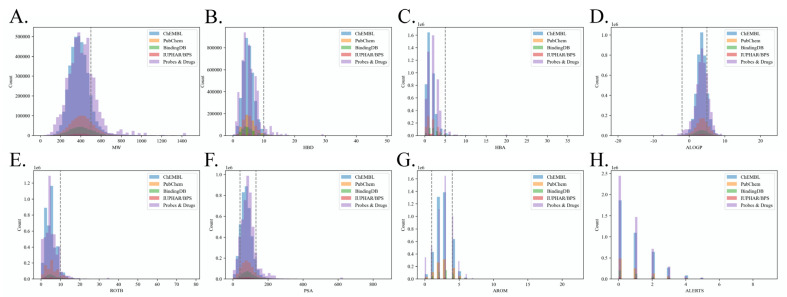
QED property distributions of benchmark dataset. (**A**) Distribution of MW; (**B**) Distribution of Num. HBD; (**C**) Distribution of Num. HBA; (**D**) Distribution of AlogP; (**E**) Distribution of Num. ROTB; (**F**) Distribution of PSA; (**G**) Distribution of Num. AROM; (**H**) Distribution of Num. ALERTS.

Hence, eight physiochemical and structural properties were calculated for all molecules in the benchmark dataset to explore the distributions of Linpski rules [34] and Quantitative Estimate of Drug-likeness (QED) [35]. These properties include molecular weights (MW), number of hydrogen bond donors (HBD), number of hydrogen bond acceptors (HBA), AlogP, number of rotatable bonds (ROTB), polar surface areas (PSA), number of aromatic rings (AROM), number of alert structures (ALERTS). The distributions of these properties in different source databases are shown in Figure 2. According to the results, approximately 83.03% of molecules’ MWs are lower than 500 Dalton (Da), with an average MW of 405; 97.89% of molecules have lower than 10 HBDs; 97.89% of molecules have lower than 5 HBAs; 82.66% of molecules’ AlogP are lower than 5, with an average AlogP of 3.49; 94.46% of molecules’ have lower than 10 ROTBs; 81.05% of molecules’ PSA obey the QED rule; 89.43% of molecules have lower than 4 and larger than 1 AROMs; 76.27% of molecules’ have lower than 1 ALERTS. Therefore, our benchmark roughly fulfills the requirements of Linpski and QED rules for drug-like molecules in HBD, HBA, ROTB, and AROM properties. However, about 20% of collected molecules are out-of-bag of Linpski and QED rules in MW, AlogP, PSA, and ALERTS properties. All of these properties should been taken into account to comprehensively estimate drug-likeness, and a study has recently observed that many approved drugs exceed the MW and HBD range outlined in these rules [36]. Meanwhile, a study claimed that the QED rule only quantifies common properties of known drugs but ignores the property differences between drug molecules and non-drug molecules [37]. As enough bio-inactive endpoints also significantly contribute to the predicting performance of LBVS models, we retained all molecules that may partly fail to comply with the Linpski and QED rules to provide negative endpoints and chemical spaces for LBVS model training and drug-like candidate identification.

### 2.2. Model Performance Comparison

We then trained EquiVS and 10 baseline models on our organized bioactivity benchmark dataset to compare the overall performances of these LBVS bioactivity prediction models, which were listed in Table 3. The results showed that EquiVS outperformed other baseline methods and achieved optimal performances on 3 metrics. Especially on MSE, the relative improvement of EquiVS compared with the suboptimal method (GBDT_ECFP) is 13.33%, while the improvements are even larger when compared to other deep learning-based methods. Meanwhile, EquiVS also performed stabler than these deep learning methods, gaining lower standard variations. The above results indicated that EquiVS achieves competitive performances in bioactivity prediction tasks, which could be used as a promising LBVS method to discover potentially active molecules for specific targets.

**Figure 3:**
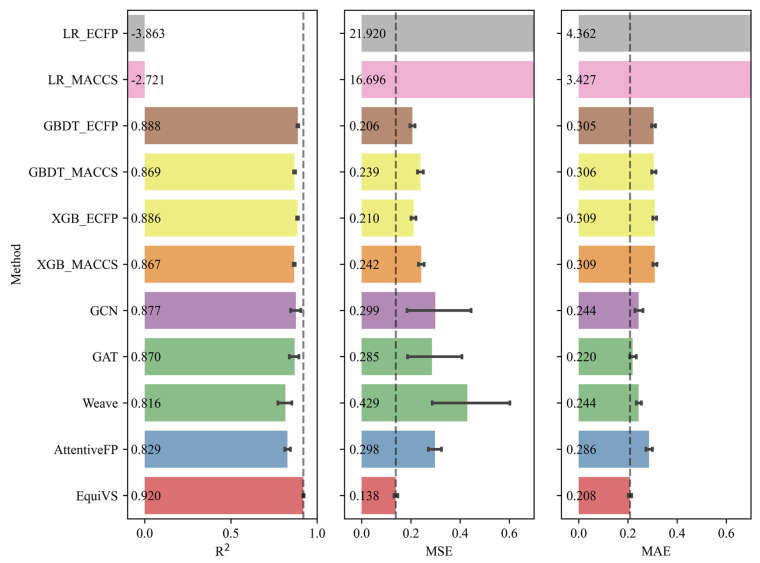
Model performances (R^2^, MSE, and MAE) of EquiVS and 10 baseline methods on the bioactivity benchmark dataset of feasible targets.

Furthermore, as the qualities and confidences of bioactivity endpoints varied in different bioactivity sub-dataset, there are a proportion of bioactivity sub-datasets that failed to be used to train bioactivity prediction models with sufficient performances. Therefore, it is necessary to exclude low-quality bioactivity sub-datasets corresponding to infeasible targets and focus on those feasible ones which have sufficient bioactivity data with fewer noises and better consistencies. Considering this, we set $R^{2}\geq 0.7$ as the threshold and filtered 1702 (82.98%) feasible targets and their bioactivity sub-datasets. The performances of EquiVS and 10 baseline methods on such feasible targets were shown in Figure 3. The results suggested that EquiVS showed a more significant superiority on good-quality targets. Compared to methods with suboptimal performances (R^2^: GBDT_ECFP, MSE: GBDT_ECFP, MAE: GAT), the relative improvements of R^2^, MSE, and MAE of EquiVS reached 3.60%, 33.01%, and 5.45%, respectively. Also, the performances of EquiVS are generally stabler than other deep learning methods.

The overall model performance comparison experiments emphasized that our designed EquiVS method can give relatively accurate predictions on molecular bioactivities rather than widely used machine learning and deep learning-based baseline methods. Especially in the scenario of bioactivity prediction with good-quality training data, the superiority of EquiVS is highlighted.

### 2.3. Ablation Study

After the effectiveness of EquiVS on large-scale bioactivity prediction has been proven, we then explored the potential mechanisms and core modules that play important roles in the molecular representation learning and model performances of EquiVS through an ablation study. Specifically, we designed five variant models, and each of them deleted or replaced a core module in EquiVS and left other modules. These variant models are as follows:

EquiVS-Single: EquiVS architecture which adopts one conformer for each molecule for structural representation learning.
EquiVS-w/o GCN: EquiVS architecture which replaces the GCN layer with a simple linear layer.
EquiVS-w/o Skip: EquiVS architecture which deletes the skip connection between different EGNN layers.
EquiVS-w/o AA: EquiVS architecture which replaces the attention-based conformer representation aggregation process with a simple sum calculation.
EquiVS-w/o IP: EquiVS architecture which deletes the conformer-level predictor and the corresponding conformer-level prediction loss.

**Figure 4:**
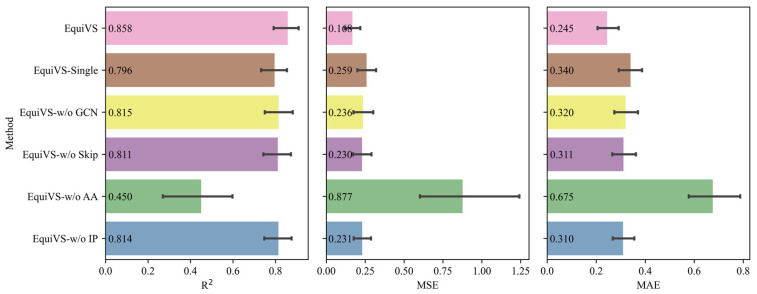
Model performances (R^2^, MSE, and MAE) of EquiVS and 5 variant models on a selective set of bioactivity benchmark dataset.

After these variants were defined, considering the running time and computing complexity, we randomly selected 50 targets and their bioactivity sub-datasets and trained EquiVS and these variants on them for performance comparisons. The data splitting settings are the same as those in model performance comparison experiments. The performances of EquiVS and its 5 variants were shown in Figure 4. The results show that EquiVS achieved optimal performances on all metrics. In addition, compared EquiVS with each of the variants, we can summarize the following five observations:

Compared to EquiVS-Single: EquiVS gained relative improvements of 7.79% on R^2^, 35.14% on MSE, and 27.94% on MAE. The results indicated that multiple sampling of molecular conformers can enhance molecular representation learning, and it is easier than a single conformer to obtain the right conformer which matches the bioactivity endpoint best, thus “diluting” the potential molecular three-dimensional structural noises caused by unsupervised conformer generation methods, which is also supported by a previous study [32].

Compared to EquiVS-w/o GCN: EquiVS gained relative improvements of 5.28% on R^2^, 28.81% on MSE, and 23.44% on MAE. The results proved that topological molecular representations learned by GCN are better than initial molecular features. Meanwhile, the effectiveness of combining two-dimensional topological level features and three-dimensional structural level features for comprehensive molecular representations.

Compared to EquiVS-w/o Skip: EquiVS gained relative improvements of 5.80% on R^2^, 26.96% on MSE, and 21.22% on MAE. The results implied that the “over-smoothing” phenomenon caused by stacking multiple EGNN layers could decrease the model performance. The use of the skip connection process can alleviate the negative impact to some degree.

Compared to EquiVS-w/o AA: EquiVS gained relative improvements of 90.67% on R^2^, 80.84% on MSE, and 63.70% on MAE. Such vastly different performance results indicated that although the fusion of multiple molecular conformer coordinates can supplement molecular three-dimensional structural information, some sampled conformers do not match the current bioactivity endpoint. Therefore, massive structural noises will be introduced and model performances will drastically decrease unless an elaborate strategy is adopted to differentiate and identify different importance for multiple molecular conformers. In contrast, EquiVS weighted aggregates different conformer representations using the attention mechanism to focus on the high-confident conformers thus benefiting the molecular representation learning and model performances.

Compared to EquiVS-w/o IP: EquiVS gained relative improvements of 5.41% on R^2^, 27.27% on MSE, and 20.97% on MAE. The results indicated that conformer-level prediction and the corresponding attention-weighted conformer-level loss could effectively assist model training and improve model performances.

### 2.4. Case Study: Optimal Molecular Conformer Discovery

**Figure 5:**
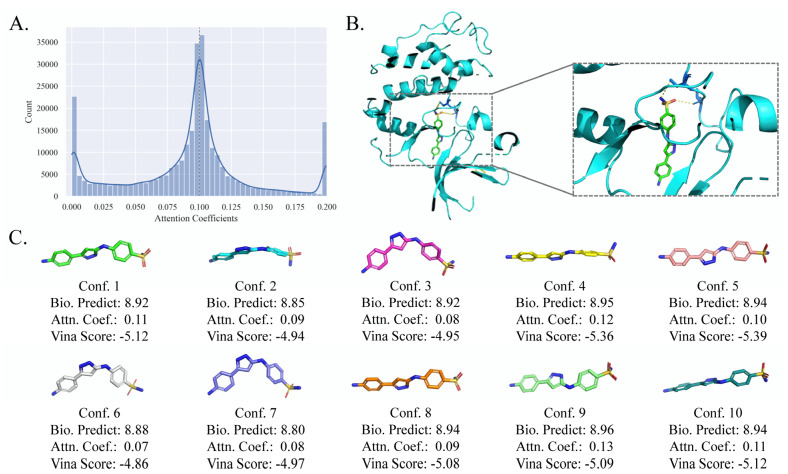
An attention visualization case for optimal conformer discovery. (**A**) Distribution of attention coefficients for conformers in CDK2 sub-dataset; (**B**) Docking pose of CDK2 complexed with the case molecule; (**C**) 10 conformers of the case molecule with bioactivity predictions, attention coefficients, and vina scores.

In EquiVS, the attention mechanism is adopted to calculate the attention coefficients of multiple molecular conformers and weighted aggregate the conformer representations. As the attention coefficients represent the importance of the molecule conformers to specific bioactivities, they can be used to interpret which conformer matches the current bioactivity and the actual molecule crystal structure best when there is a ligand-protein binding. To investigate the potential of EquiVS in optimal molecular conformer discovery, we took human cyclin-dependent kinase 2 (CDK2) as a candidate target and its bioactivity sub-dataset to visualize all attention coefficients (Figure 5A). The results showed that quite a few attention coefficients generated by EquiVS are close to 0, indicating that there are noises in some calculated molecular conformers. Representing the molecule structures with single conformers or not differentiating the reliabilities of multiple conformers could lead to a negative impact on the correctness and effectiveness of molecular structural representations. Combining the result that a proportion of attention coefficients are significantly larger than the average (0.10), the overall distribution visualization indicated that EquiVS can recognize and distinguish different molecular conformers to reduce the influence of structurally unreasonable conformers on the bioactivity predictions.

Moreover, we selected “Nc1ccc(-c2cc(Nc3ccc(S(N)(=O)=O)cc3)[nH] n2)cc1” as a case active molecule (bioactivity value: 9.0 -logM), visualized its conformers, predicted the bioactivity values by the conformer-level predictor in EquiVS, generated the binding score and pose by Autodock Vina [38], to investigate the consistency of attention coefficients, predicted bioactivities, and vina scores. The binding pose of CDK2 and the case molecule was shown in Figure 5B, showing that molecular docking successfully discover two binding sites for the case molecule. Then, the molecular conformers, attention coefficients, predicted bioactivities, and vina scores were shown in Figure 5C. It is inspiring to discover the observation that there is kind of consistencies and correlations between attention coefficients and the accuracy of predicted bioactivities and vina scores. That is, the conformers with higher attention coefficients tend to get more accurate bioactivity predictions and higher vina scores. For instance, conformer 4 with the second biggest attention coefficient achieved the second most accurate prediction and Vina score. Also, the prediction accuracies and vina scores of those with attention coefficients below the average (conformer 2, 3, 6, 7, and 8) are much lower than the else.

The overall results suggested that the attention-based conformer-level model interpretability of EquiVS can differentiate between molecular conformers with varied structure reliabilities and discover the optimal conformers for identifying specific bioactivity endpoints and targets. Compared to the machine learning-based LBVS methods, which could not directly handle molecular 3D structures and lacked interpretabilities, the attention mechanism in EquiVS provides valuable insights regarding the optimal conformers and discovery of binding poses.

## 3. Materials and Methods

### 3.1. Bioactivity Data Collecting, Integrating and Filtering

**Figure 6:**
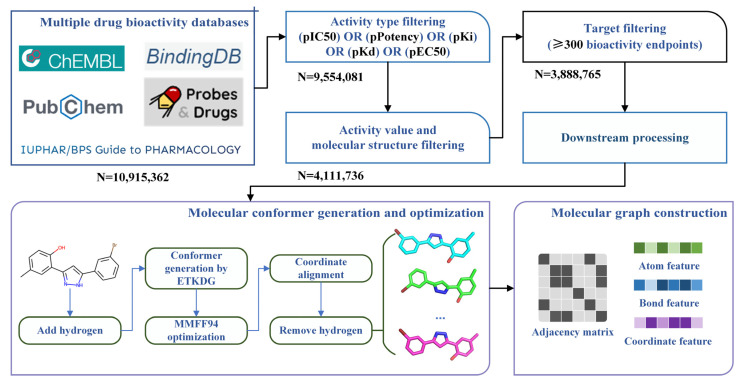
Bioactivity data collecting, integrating, filtering and processing workflow.

We introduced the data collection, integration, filtering, and preprocessing process of the molecular bioactivity benchmark dataset for drug virtual screening in detail. Figure 6 shows the overall process of the construction of the benchmark dataset, including (1) Collection and integration of multi-source drug bioactivity data; (2) Multilevel data filtering based on activity type, activity unit, molecular structure, and target; (3) Downstream processing, including molecular conformer generation and optimization, and molecular graph construction.

In the first steps, we collected large-scale molecular bioactivity data from five public databases, including ChEMBL [39], PubChem [1], BindingDB [40], Probes and Drugs [41], and IUPHAR/BPS [42]. We directly downloaded the integrated data of the above five databases from a previous study [33] to accelerate the data collection process. Brief descriptions of the introduced databases are listed in Table 4. The collected bioactivity data derived from [33] have nine items, including (1) molecule ID corresponding to the source database, (2) molecule name, (3) molecule structure represented using SMILES (Simplified Molecular Input Line Entry Specification), (4) HGNC (HUGO Gene Nomenclature Committee) [43]-named target ID, (5) bioactivity type, (6) bioassay type, (7) Bioactivity unit, (8) bioactivity value, and (9) data source.

As for the data integration, in [33], the bioactivity data have been preprocessed, which first identified matched molecules from different sources and integrated their relevant bioactivity data, then labeling “1 structure”, “match”, “no structure”, or specific Tanimoto structure similarity based on Morgan fingerprints [44] for each integrated data. Furthermore, the corresponding bioactivity values have been integrated too. Specifically, a set of buckets were set determined by negative decadic logarithm with a molar unit (e.g., a bucket with the range of 5 -logM to 6 -logM). Then, different bioactivity values to a matched molecule were classified into the corresponding buckets based on their ranges. The average value in each bucket was calculated. In our study, we selected the “1 structure” and “match” labeled bioactivity data to allow structural consistency, and the average values of the buckets with the highest frequency as the final bioactivity values.

In the second step, we proposed a multilevel data filtering strategy to identify high-quality bioactivity data. For bioactivity data, we selected endpoints with five widely used and studied activity types (IC50, Ki, Kd, EC50, and Potency) as candidates and filtered the last ones. For molecular structure, we adopted MolVS [45] for structure standardization, including (1) Normalization of functional groups to a consistent format; (2) Recombination of separated charges; (3) Breaking of bonds to metal atoms; (4) Competitive reionization to ensure strongest acids ionize first in partially ionize molecules; (5) Tautomer enumeration and canonicalization; (6) Neutralization of charges; (7) Standardization or removal of stereochemistry information; (8) Filtering of salt and solvent fragments; (9) Generation of fragment, isotope, charge, tautomer or stereochemistry insensitive parent structures; (10) Validations to identify molecules with unusual and potentially troublesome characteristics. Then, the mistake molecules which cannot be identified by RDkit package [46] were filtered. For biological targets, as the prediction performances of machine learning-based and deep learning-based LBVS methods highly rely on the quality and quantity of bioactivity data for specific targets, we only selected those targets with more than 300 bioactivity endpoints as candidates and filtered the corresponding bioactivity data of other targets. Finally, 3,888,765 bioactivity endpoints with 954 targets were left. Regarding bioactivity endpoints belonging to a specific target as a bioactivity sub-dataset, we gathered these 954 sub-datasets together and assembled them into a bioactivity benchmark dataset.

### 3.2. Molecular Conformer and Graph Generating

Candidate bioactivity endpoints were determined by the above comprehensive process. However, these data contained only one-dimensional SMILES representations of molecular structures and lacked three-dimensional structural information. To address this issue, multiple molecular conformers were computationally generated and assembled with bioactivity endpoints to finish the bioactivity benchmark dataset construction. 

In this study, we used the RDKit package to generate and optimize molecular conformers. Specifically, hydrogen atoms were first added to the molecular structure to simulate the real molecular geometric conformation. Then, ETKDG (Experimental-Torsion basic Knowledge Distance Geometry) was used for conformation generation. ETKDG is a knowledge-based conformer generation method that combines distance geometry and torsion angle preferences proposed by Riniker et al [47]. Additionally, since a molecule could have multiple three-dimensional conformers at different chemical environments, we used ETKDG methods with different random initializations to generate 10 conformers for each molecule to achieve sufficient sampling of molecule three-dimensional structures. To better approximate the actual geometric conformations of molecules, we optimized the generated molecular conformers using MMFF94 force field, which is developed by Merck that uses multiple empirical parameters, including atomic types, charges, bond lengths, bond angles, torsional angles, van der Waals force, etc., to represent the potential energy and optimize the molecular conformation to finally obtain approximately optimal low-energy conformers [48].

Here, we aligned the three-dimensional coordinates of different conformers of each molecule to normalize coordinate information. All hydrogen atoms were removed from the molecular structures to reduce the computing complexity and training time of LBVS methods. Finally, we assembled all conformers to corresponding molecule SDF structure files to finish the construction of the large-scale bioactivity benchmark dataset.

**Figure 7:**
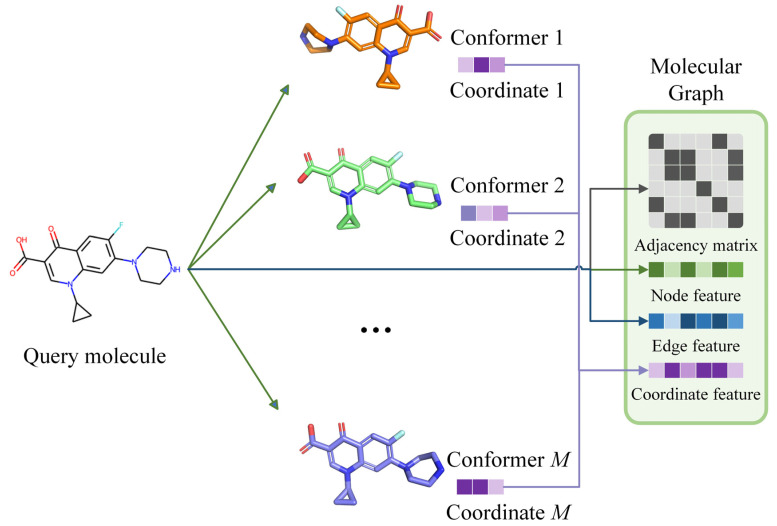
Diagram of constructing a molecular graph from the query molecule and its conformers.

To introduce the molecular conformer information to our GNN-based EquiVS method, we constructed each molecular graph with an adjacency matrix, a node feature matrix $H_{V}$, an edge feature matrix $H_{ℰ}$, and a coordinate feature matrix $H_{C}$, where an example of the representations of molecules in our study was shown in Figure 7. 

Given a molecule, its graph G can be represented as:

(1) G=(V,ℰ)

where V denotes the set of atoms, and ℰ denotes the set of bonds. The topological structure of G can be represented as an adjacency matrix A. Given an atom node i, its neighbor atom node j, and its neighbor node set $N_{i}$, the adjacency relation A(i,j) can be represented as:

(2) $$A\left(i,j\right)=\{\begin{array}{c} 1,\;j\in N_{i}\\ 0,\;j\notin N_{i} \end{array}$$

As for feature matrices in the molecular graph, we first calculated atom physiochemical properties as $H_{V}$ with the dimension of 74, including (1) one-hot encoding of atomic elements; (2) one-hot encoding of atomic degrees; (3) one-hot encoding of the number of implicit hydrogens; (4) one-hot encoding of the formal charges; (5) one-hot encoding of the number of radical electrons; (6) one-hot encoding of the atom hybridizations; (7) one-hot encoding of the aromatics; (8) one-hot encoding of the number of total hydrogens. Then, the chemical bond properties were adopted as $H_{ℰ}$ with the dimension of 12, including (1) one-hot encoding of bond types; (2) conjugation; (3) ring; (4) one-hot encoding of the stereo configuration. Finally, we collected the atomic spatial coordinates in each conformer and concatenated them as $H_{C}$ with a dimension of 30. Through the above processes, the molecular three-dimensional structural information was represented in molecule graphs, which can be identified in EquiVS.

### 3.3. Bioactivity Prediction Model Construction

**Figure 8:**
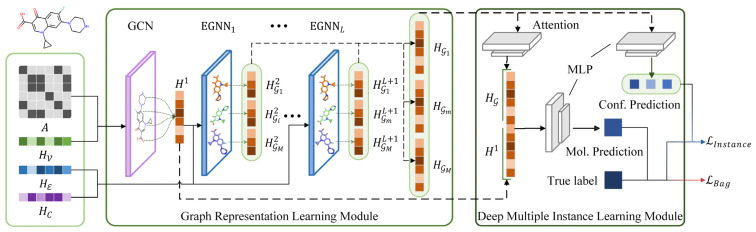
The architecture of EquiVS.

In this study, we proposed an EGNN and deep MIL-based virtual screening method, EquiVS, to facilitate the representation learning of current LBVS methods via utilizing molecular conformations. The architecture of EquiVS (Figure 8) comprises two core modules: the graph representation learning module and the deep multiple instance learning module. We introduced the computing flow of these modules in detail.

***Graph representation learning module***. We considered learning molecule high-order representations from both two-dimensional topological level and three-dimensional structural level. Therefore, a stepwise molecular graph learning strategy with skip connection was designed to learn and aggregate the molecular representations. Specifically, a GCN layer is first used to learn graph topological representations $H^{1}$, which can be represented as:

(3) $$h^{1}=GCN\left(A,h_{V},W^{GCN}\right)$$

where $h^{1}$, $h_{V}$ are learned node features and initial node features, respectively. $W^{GCN}$ is a trainable parameter matrix. Considering the message passing process in GCN, given a target node i and one of its neighbor nodes j, the message from j is:

(4) $$m_{ij}=\phi_{e}\left(h_{V_{i}},h_{V_{j}}\right)$$

where $\phi_{e}$ is a linear transformation function. Then, GCN aggregates the message and the input feature $h_{C_{G_{m}},i}^{l}$ to finish node feature updating:

(5) $$h_{i}^{1}=\phi_{h}\left(h_{V_{i}},\sum_{j\in N_{i}}m_{ij}\right)$$

where $\phi_{h}$ is a linear transformation function. Then, a readout function is further adopted to aggregate node feature $h^{1}$ to graph feature $H^{1}$:

(6) $$H^{1}=\sum \left(\sigma \left(W^{READOUT_{1}}h^{1}+b^{READOUT_{1}}\right)\cdot h^{1}\right)$$

where σ is Sigmoid activation function, $W^{READOUT_{1}}$ and $b^{READOUT_{1}}$ are trainable parameter matrices. Then, L EGNN layers were used to learn structural representations for each conformer. Given M conformers for a specific molecule, by dividing its coordinate feature matrix into M matrices, the overall graph G can be represented as:

(7) $$G=\left\{G_{m}|i\leq M\right\}$$

where m is a given molecular conformer. Taking l-th EGNN layer as an example, the molecule representations of each $G_{m}$ is learned through:

(8) $$h_{G_{m}}^{l+1}=EGNN_{l}\left(A,\;h_{V_{G_{m}}}^{l},h_{{ℰ}_{G_{m}}}^{l},h_{C_{G_{m}}}^{l},W^{l+1}\right)$$

where $h_{V_{G_{m}}}^{l}$, $h_{{ℰ}_{G_{m}}}^{l}$, and $h_{C_{G_{m}}}^{l}$ are node feature, edge feature, and coordinate feature in l-th EGNN layer, respectively. Considering the message passing process in $EGNN_{l}$, given a target node i and one of its neighbor nodes j, the message from j is:

(9) $$m_{ij}=\phi_{e}\left(h_{V_{G_{m}},i}^{l},h_{V_{G_{m}},j}^{l},\|h_{C_{G_{m}},i}^{l}-h_{C_{G_{m}},j}^{l}\|{}^{2},\;h_{{ℰ}_{G_{m},ij}}^{l}\right)$$

where $\phi_{e}$ is a linear transformation function. Meanwhile, the coordinate features are also updated in EGNN:

(10) $$h_{C_{G_{m}},i}^{l+1}=h_{C_{G_{m}},i}^{l}+C\sum_{j\in N_{i}}\left(h_{C_{G_{m}},i}^{l}-h_{C_{G_{m}},j}^{l}\right)\phi_{x}\left(m_{ij}\right)$$

where C is the number of neighbor nodes minus 1, and $\phi_{x}$ is a linear transformation function. Finally, EGNN aggregates the message and the input feature $h_{C_{G_{m}},i}^{l}$ to finish node feature updating:

(11) $$h_{i}^{l+1}=\phi_{h}\left(h_{i}^{l},\sum_{j\in N_{i}}m_{ij}\right)$$

where $\phi_{h}$ is a linear transformation function After the node features are updates through the above message passing process, similarly, a readout function is used to generate graph feature $H_{G_{m}}^{l+1}$ or $G_{m}$:

(12) $$H_{G_{m}}^{l+1}=\sum \left(\sigma \left(W^{READOUT_{l}}h_{V_{G_{m}}}^{l}+b^{READOUT_{l}}\right)\cdot h_{V_{G_{m}}}^{l}\right)$$

Finally, as the “over-smoothing” and “vanishing gradient” widely exist in deep graph neural network models, which could significantly harm the model performances, we built skip connections between different EGNN layers to allow direct gradient propagation to the shallow layers by simply concatenating the graph feature output of each layer as the final graph representations. For conformer $G_{m}$, its final graph feature $H_{G_{m}}$ can be formulated as:

(13) $$H_{G_{m}}=Concat(H_{G_{m}}^{2},H_{G_{m}}^{3},\ldots ,H_{G_{m}}^{L+1})$$

***Deep multiple instance learning module***. After EquiVS captures the high-order representations for molecular conformers, the aggregation process of these conformer representations to generate comprehensive molecular representations, and effective bioactivity prediction through both conformer-level and molecule-level should be elaborately considered and designed. Regarding this, we introduced MIL theory into bioactivity prediction and designed our deep multiple instance learning module with an interpretable attention mechanism. Based on MIL theory, a molecule can be regarded as a “bag”, and its multiple conformers are “instances”. The bioactivity value is only labeled at the molecule level, but the conformer-level bioactivities still remain unknown. Therefore, the training object of MIL is to accurately predict the molecular bioactivity value, while identifying the conformer which fits this bioactivity value best, simultaneously. Regarding this, EquiVS first dynamically aggregates the conformer instance representations with an attention mechanism. The attention score of $H_{G_{m}}$ can be formulated as:

(14) $$w_{G_{m}}=q^{T}\cdot \sigma \left(W^{Attn}\cdot H_{G_{m}}+b^{Attn}\right)$$

where σ is a Tanh activation function. q, $W^{Attn}$, and $b^{Attn}$ are trainable parameter matrices. The attention score can be further converted to normalized attention coefficient $\alpha_{G_{m}}$:

(15) $$\alpha_{G_{m}}=\frac{exp\left(w_{G_{m}}\right)}{\sum_{m=1}^{M}exp\left(w_{G_{m}}\right)}$$

The attention coefficient represents the importance weight of a conformer. Hence, the conformer instance representations can be aggregated to acquire molecule representation $H_{G}$ based on the attention coefficients:

(16) $$H_{G}=\sum_{m=1}^{M}\alpha_{G_{m}}H_{G_{m}}$$

Then, EquiVS predicts the bioactivity value from both conformer-level and molecule-level. For conformer-level prediction, a multilayer perceptron (MLP) is used for bioactivity prediction:

(17) $$y_{G_{m}}=W_{2}^{Ins}\sigma \left(W_{1}^{Ins}\left(H_{G_{m}}\right)+b_{1}^{Ins}\right)+b_{2}^{Ins}$$

where σ is a ReLU activation function. $W_{1}^{Ins}$, $b_{1}^{Ins}$, $W_{2}^{Ins}$, and $b_{2}^{Ins}$ are trainable parameter matrices. For molecule-level prediction, another MLP is constructed:

(18) $$y_{G}=W_{2}^{Bag}\sigma \left(W_{1}^{Bag}\left(Concat\left(H^{1},H_{G}\right)\right)+b_{1}^{Bag}\right)+b_{2}^{Bag}$$

where σ is a ReLU activation function. $W_{1}^{Bag}$, $b_{1}^{Bag}$, $W_{2}^{Bag}$, and $b_{2}^{Bag}$ are trainable parameter matrices. Based on the above processes, EquiVS achieves molecular three-dimensional structure-based bioactivity prediction.

### 3.4. Training Optimization

Inspired by loss-based deep multiple instance learning [49], we adopted both conformer-level prediction and molecule-level prediction to calculate model loss and update model parameters. Meanwhile, we also differentiated the conformer predictions by attention coefficients, thus alleviating the impact of noisy conformers for model training. Specifically, we introduced mean square error (MSE) as the optimization function. Given N samples as the batched data, the conformer-level prediction loss ${ℒ}_{Ins}$ can be formulated as:

(19) $${ℒ}_{Ins}=\frac{1}{N}\sum_{n=1}^{N}\sum_{m=1}^{M}\alpha_{G_{n,m}}{\left(y_{n}-{\hat{y}}_{G_{n,m}}\right)}^{2}$$

where $\alpha_{G_{n,m}}$ is the attention coefficient for m-th conformers in n-th molecule. $y_{n}$ and ${\hat{y}}_{G_{n,m}}$ are the true label for n-th molecule and predicted label for m-th conformers in n-th molecule, respectively. The molecule-level prediction loss ${ℒ}_{Bag}$ can be formulated as:

(20) $${ℒ}_{Bag}=\frac{1}{N}\sum_{n=1}^{N}{\left(y_{n}-{\hat{y}}_{n}\right)}^{2}$$

where ${\hat{y}}_{n}$ is the molecule-level predicted label for n-th molecule. The final loss function can be represented as:

(21) $$ℒ=\beta {ℒ}_{Ins}+\left(1-\beta \right){ℒ}_{Bag}$$

where β is a contribution factor to determine the importance of ${ℒ}_{Ins}$ and ${ℒ}_{Bag}$. In this study, we set β as 0.5.

The overall EquiVS computing flow was shown in Algorithm 1. Furthermore, we used Adam optimizer for training optimization and added a dropout layer after each GNN layer and MLP layer to alleviate overfitting.
**Algorithm 1**: The computing flow of EquiVS**Input**: A molecule graph G, the set of atoms V, the set of bonds ℰ. Its node feature $H_{V}$, edge feature $H_{ℰ}$, and coordinate feature $H_{C}$. A set of molecular conformation $\left\{G_{i}|i\leq M\right\}$ for G.**Output**: Predicted bioactivity $y_{G}$ for G on a specific target.  1: Initialize the trainable parameters in EquiVS.   2: Acquire 2D topological graph representation $H^{1}\leftarrow GCN\left(H_{V}\right)$ by **Equation (3)**.   3: **for** *Molecular conformers* $G_{m}\leftarrow G_{1},\ldots ,G_{M}$ **do**
  4:    **for** *EGNN layer*
l←1,…,L **do**
  5:        Acquire 3D structural graph representation       $H_{G_{m}}^{\left(l+1\right)}\leftarrow EGNN\left(H_{V_{G_{m}}}^{l},H_{{ℰ}_{G_{m}}}^{l},H_{C_{G_{m}}}^{l}\right)$ by **Equation (8)**.   6:   **end for**
  7:   Concatenate the conformer representation $H_{G_{m}}$ by **Equation (13)**.   8: **end for**
  9: Acuiqre aggregated graph representation with attention mechanism     $\alpha_{G_{m}},H_{G}\leftarrow Attention\left(\left\{H_{G_{m}}|m\leq M\right\}\right)$ by **Equations (14)–(16)**. 10: Generate conformer-level prediction $y_{G_{m}}\leftarrow MLP\left(H_{G_{m}}\right)$ by **Equation (17)**. 11: Generate molecule-level prediction $y_{G}\leftarrow MLP\left(H^{1},H_{G}\right)$ by **Equation (18)**. 12: Calculate conformer-lebel loss ${ℒ}_{Ins}$ and molecule-level loss ${ℒ}_{Bag}$ by **Equations (19) and (20)**. 13: Update parameters by optimizing **Equation (21)**. 14: Ouput $y_{G}$.

### 3.5. Model Interpretation

As described in *Deep multiple instance learning module*, EquiVS employs the attention mechanism to aggregate the conformer representations, which could also be used for conformer-level model interpretation. Given a bioactivity endpoint with a molecule and multiple conformers, we ranked the importance of different conformers based on the attention coefficients $\left\{\alpha_{G_{i}}|i\leq M\right\}$ to discover the optimal conformer which matches the current bioactivity value best.

### 3.6. Settings

For model hyper-parameter settings, we set the learning rate as 0.001, the dropout rate as 0.05, the number of epochs as 200, batch size as 128, the hidden feature dimensions as 128, and the number of EGNN layers as 2 to construct and train EquiVS on our proposed bioactivity prediction benchmark dataset.

For baseline settings, we adopted two molecular fingerprints (ECFP4 [44] and MACCS [50]), three 3 machine learning methods (Linear regression LR, gradient boosting decision tree GBDT [51], and extreme gradient boosting decision tree XGB [52]), and four GNN methods (GCN [53], GAT [54], AttentiveFP [55], and Weave [56]) to construct baseline models to perform model performance comparisons.

For experimental settings, we randomly split the benchmark dataset into training set, validating set, and testing set with a ratio of 8:1:1. EquiVS and all baseline methods were trained on the training set, adjusting hyper-parameters on the validating set, and evaluated on the testing set. We adopted the coefficient of determination (R^2^), MSE, and mean absolute error (MAE) to assess the performances of bioactivity prediction. These metrics can be formulated as:

(22) $$R^{2}=1-\frac{\sum_{i}{\left(y_{i}-\hat{y_{i}}\right)}^{2}}{\sum_{i}{\left(y_{i}-\bar{y_{i}}\right)}^{2}}$$

(23) $$MSE=\frac{1}{N}\sum_{i=1}^{N}{\left(y_{i}-\hat{y_{i}}\right)}^{2}$$

(24) $$MAE=\frac{1}{N}\sum_{i=1}^{N}\left|y_{i}-\hat{y_{i}}\right|$$

where N denotes the sample size. $y_{i}$, $\hat{y_{i}}$, and $\bar{y_{i}}$ are true label, predicted label, and overall average label.

## 4. Conclusions

In this study, we investigate the role of molecular conformation in LBVS and bioactivity prediction scenarios. A large-scale bioactivity prediction benchmark dataset is proposed to assemble the requirement of molecular conformation to bioactivity endpoints, which is collected from multiple public pharmaceutical databases and contains thousands of targets and millions of bioactivity endpoints, molecules, and molecule conformers. Then, an EGNN and deep MIL-based LBVS method is designed for bioactivity prediction, which is called EquiVS. Compared to other widely-used ML-based and GNN-based methods, EquiVS achieved notable improvements on our large-scale benchmark dataset. Combining the ablation analysis, the performance results prove employing molecular conformation could enhance molecular representation learning and further contribute to better bioactivity prediction with elaborate neural network architecture design and reasonable feature extraction and aggregation. To promote the practical application of EquiVS, two case studies are designed to explore the effectiveness of conformer-level interpretation in EquiVS. The overall results reveal a promising prospect of molecular conformation as well as our proposed benchmark dataset and EquiVS method in bioactivity prediction and LBVS.

It should also be emphasized that there are several major limitations in our study. First, from the data quality control aspect, some of the sub-datasets and targets in our integrated benchmark dataset should be filtered as all tested LBVS methods could not give reliable predictions on them, but they are still retained in the current version; Second, from the model training aspect, a predictable optimization is to pre-train EGNN-based models using molecular conformation and finetune them for the downstream bioactivity prediction [57,58,59]. Also, improving the GNN model training period with chemical domain knowledge insights is another promising strategy [60,61]. Thirdly, based on the architecture design aspect, more advanced GNN backbones that use 3D graphs as inputs should be adopted for conformer-based representation learning, such as SchNet [62], GemNet [63], PaiNN [64], etc.

As for future research, we will focus on developing practical tools to enhance the accessibility of EquiVS and employing molecular conformation using massive unlabeled molecules to pre-train and optimize EquiVS models in terms of robustness and generality.

## Figures and Tables

**Table 1:** A list of deep learning-based LBVS methods.

Name	Method	Feature	Compared LBVS Methods
WDL-RF [22]	Convolutional neural network and random forest	Molecular atomic properties	Random forest-based LBVS methods with NGFP, MACCS, ECFP2, ECFP4, ECFP8, ECFP10, FCFP2, FCFP4, FCFP8, and FCFP10 fingerprints.
DeepScreening [19]	Deep neural network	Multiple molecular fingerprints extracted from 2D molecular structures	-
SED [23]	Deep neural network	Molecular ECFP fingerprints extracted from 2D molecular structures	Different length of ECFP fingerprints and multiple machine learning-based LBVS methods (GBDT, SVR, and RF)
Winter et al. [24]	Recurrent neural network and convolutional neural network	Tokenized molecular representations from 1D molecular sequences	Similarity-based LBVS methods with lAval, TT, lECFP4, lECFP6, ECFP4, RDK5, Avalon, ECFP6, FCFP4, MACCS, FCFC4, and ECFC0 fingerprints.
GATNN [20]	Graph attention network	2D molecular graphs	Similarity-based LBVS methods with ECFP4, TT, MHFP6, and ECFC0 fingerprints.
RealVS [21]	Graph attention network	2D molecular graphs	Multiple graph neural network-based LBVS methods (GCN, GAT, GIN, NeuralFP, Weave, MPNN, WDL-RF, and AttentiveFP).
Altalib et. al. [25]	Hybrid Siamese convolutional neural network	Molecular ECFC fingerprints extracted from 2D molecular structures	Similarity-, Bayesian inference-, deep belief network-, and single Siamese convolutional neural network-based LBVS with ECFC4 and ECFP4 fingerprints.

Abbreviations: NGFP: neural graph fingerprints; MACCS: molecular access system fingerprints; ECFP: extended-connectivity fingerprints; FCFP: functional-class fingerprints; GBDT: gradient-boosting decision tree; SVR: support vector regression; RF: random forest; lAval: long Avalon fingerprints; TT: topological torsion fingerprints; lECFP: long ECFP fingerprints; RDK: RDKit fingerprints; FCFC: feature-connectivity count vector fingerprints; ECFC: extended-connectivity count vector fingerprints; MHFP: MinHash fingerprints; GCN: graph convolutional network; GAT: graph attention network; GIN: graph isomorphism network; NeuralFP: neural fingerprint; MPNN: message-passing neural network; WDL-RF: weighted deep learning and random forest.

**Table 2:** Descriptive statistical results of bioactivity endpoints in different source databases.

Bioactivity	Statistics	ChEMBL	PubChem	BindingDB	IUPHAR/BPS	Probes&Drugs
Value	Avg.	5.33	6.29	6.17	7.31	5.52
	Std.	1.25	1.52	1.58	1.48	1.48
	Min.	0.10	0.10	0.10	0.80	0.10
	Max.	15.90	13.00	12.20	18.00	21.80
Category	Num. Active	939,036	540,384	228,907	78,483	1,656,557
	PCT. Active	23.56%	56.72%	54.9%	81.42%	32.93%
	Num. Inactive	3,047,145	412,288	188,067	17,915	3,373,950
	PCT. Inactive	76.44%	43.28%	45.1%	18.58%	67.07%

Footnote: Avg.: Average; Std.: Standard Deviation; Num.: Number of; PCT.: Percentile of.

**Table 3:** Model performances of EquiVS and 10 baseline methods on bioactivity benchmark dataset.

Type	Method	R^2^	MSE	MAE
Machine Learning	LR_ECFP	−4.543 ± 5.443	24.844 ± 24.904	4.960 ± 4.956
	LR_MACCS	−3.438 ± 5.302	19.853 ± 24.339	4.063 ± 4.798
	GBDT_ECFP	0.831 ± 0.189	0.240 ± 0.256	0.325 ± 0.176
	GBDT_MACCS	0.810 ± 0.202	0.273 ± 0.286	0.328 ± 0.190
	XGB_ECFP	0.830 ± 0.188	0.243 ± 0.254	0.329 ± 0.175
	XGB_MACCS	0.808 ± 0.201	0.276 ± 0.285	0.331 ± 0.189
Deep Learning	GCN	0.768 ± 0.960	0.509 ± 3.868	0.280 ± 0.412
	GAT	0.760 ± 0.838	0.413 ± 2.962	0.259 ± 0.282
	Weave	0.705 ± 1.056	0.601 ± 4.051	0.286 ± 0.297
	AttentiveFP	0.746 ± 0.390	0.359 ± 0.587	0.330 ± 0.300
	EquiVS	**0.833 ± 0.243**	**0.208 ± 0.282**	**0.257 ± 0.189**

Footnote: The best results in each column are in bold faces and the second-best results are underlined.

**Table 4:** Detailed information about the five databases.

Database	Description	Version	Num. Molecule
ChEMBL	Contains the bioactivity data of more than 2.1 million experimentally determined drug-like molecules	28	1,131,947
PubChem	Contains the bioactivity data and physiochemical properties of more than 1.1 million molecules	11.01.21	444,152
BindingDB	Contains binding affinity data of approximately 26,000 drug-like molecules regarding specific biological targets	25.02.21	26,856
Probes&Drugs	Contains manually collected biological target and bioactivity data of pharmacologically active compounds	2021.1	34,211
IUPHAR/BPS	Contains bioactivity data, target, and signaling pathway information of approximately 29,000 compounds derived from 30 public and commercial libraries	02b_2021	7371

## Data Availability

The source codes of EquiVS and molecular conformer generation process and the benchmark dataset are available at https://github.com/gu-yaowen/EquiVS.

## Sample Availability

Samples of the compounds are available from the authors.
